# Internet-delivered cognitive therapy for social anxiety disorder in Hong Kong: A randomized controlled trial

**DOI:** 10.1016/j.invent.2022.100539

**Published:** 2022-04-18

**Authors:** Graham R. Thew, Amy P.L. Kwok, Mandy H. Lissillour Chan, Candice L.Y.M. Powell, Jennifer Wild, Patrick W.L. Leung, David M. Clark

**Affiliations:** a Department of Experimental Psychology, University of Oxford, UK; b Oxford University Hospitals NHS Foundation Trust, UK; c Oxford Health NHS Foundation Trust, UK; d Hong Kong East Cluster, Hospital Authority, Hong Kong, China; e New Life Psychiatric Rehabilitation Association, Hong Kong, China; f Department of Psychology, The Chinese University of Hong Kong, Hong Kong, China

**Keywords:** Social anxiety, Internet interventions, Dissemination, Cross-cultural, Cognitive behavioural therapy

## Abstract

**Background:**

Research is needed to determine the extent to which internet-delivered psychological therapies are effective when delivered in countries and cultures outside of where they were developed.

**Objective:**

This waitlist-controlled study evaluated the efficacy of a UK-developed, therapist-guided internet Cognitive Therapy programme for Social Anxiety Disorder (iCT-SAD) when delivered in Hong Kong by local therapists.

**Methods:**

Patients were randomized to iCT-SAD (n = 22) or a waitlist control group (n = 22). Assessments took place at weeks 0, 8, and 15 (posttreatment/postwait), with a further 3-month follow-up assessment for the iCT-SAD group. The primary outcome measure was the Liebowitz Social Anxiety Scale (self-report), and posttreatment/postwait diagnostic assessments were completed by independent assessors blind to condition. Trial Registration: ISRCTN11357117.

**Results:**

Compared with the waitlist group, iCT-SAD significantly reduced social anxiety symptoms (adjusted difference at posttreatment 55.36, 95%CI 44.32 to 66.39, *p* < 0.001; *d*_Cohen_ 2.41). The treatment was also superior to waitlist on all secondary outcome measures. 86% of the iCT-SAD group demonstrated remission from SAD based on the LSAS, compared to 5% of the waitlist group. 73% no longer met diagnostic criteria at posttreatment, compared to 9% of the waitlist group. The gains made by the iCT-SAD group were maintained at three-month follow-up.

**Conclusions:**

iCT-SAD showed strong efficacy for the treatment of SAD in Hong Kong. As the clinical outcomes were similar to UK studies, this suggests the dissemination of the treatment into a different cultural setting did not result in a substantial loss of efficacy.

## Highlights

• iCT-SAD was an efficacious treatment for social anxiety in Hong Kong.
• Implementation in Hong Kong obtained outcomes similar to UK studies.
• iCT-SAD required less therapist time compared to face-to-face treatment.

## 1 Introduction

One of the key benefits of online psychological interventions is their potential to be transported and delivered in locations where mental health services may be less well established, where demand is high, or in more rural or remote communities. However, the global reach of such interventions has yet to be fully realised, and research is required to assess their feasibility, acceptability, and efficacy when implemented in new contexts. There are currently few studies that examine the efficacy of online interventions when transported internationally or interculturally (Gallego et al., 2011; Jakobsen et al., 2017; Kishimoto et al., 2016; Tulbure et al., 2015). Results have generally been promising, suggesting such transportation is achievable without substantial loss of efficacy. However, as internet interventions vary widely in terms of content, format, and the extent and nature of therapist guidance, it is important to examine a wider range of individual treatments before we can consider general trends regarding international dissemination.

Internet-delivered interventions for Social Anxiety Disorder (SAD) have been extensively researched, and a range of treatments exist with empirical evidence of their efficacy. One metaanalysis (Kampmann et al., 2016) found 21 trials of internet-delivered Cognitive Behavioural Therapy (CBT) interventions, which showed a mean within-group pre-post effect size (Hedge's *g*) of 0.96, indicating a large effect on SAD symptoms. In the UK, Internet-delivered Cognitive Therapy for SAD (iCT-SAD; Stott et al., 2013) has been developed based on the Clark and Wells (1995) cognitive model and the associated face-to-face treatment protocol recommended by the National Institute for Health and Care Excellence (NICE, 2013). Results of initial studies have suggested it may be a particularly promising treatment, with large pre-post effect sizes (Cohen's *d* > 1.5) in an initial development case series (Stott et al., 2013) and in a recent randomized controlled trial (RCT) (Clark et al., submitted) where iCT-SAD showed similar efficacy to face-to-face delivery of the cognitive therapy.

However, most studies to date have been conducted in the same settings as where they were developed, meaning our understanding of how these treatments perform in other countries and cultures is limited. This is particularly true for iCT-SAD, which contains components not found in other internet SAD interventions, such as the ‘self-focused attention and safety behaviours’ experiment, where the patient converses over webcam with a stranger; and video feedback, where the patient is guided to view footage of themselves engaging in social interactions. It is not yet known whether internet therapies of this type can retain their high efficacy when implemented outside of the culture in which they were developed. The lack of ‘transportation’ studies also means we know little about the level of treatment adaptation that might be required. Frameworks for undertaking cultural adaptation have been described (Bernal et al., 2009; Hwang, 2009), but only more recently has a more empirical approach to this process been taken (Naeem et al., 2016; Rathod et al., 2019). The extent and nature of cultural adaptations made to internet interventions is generally poorly described in the literature. One approach is to start by evaluating the performance of a largely unadapted treatment, with the findings of the evaluation being used to determine whether further, culturally sensitive adaptation might be required.

The aim of this study was to assess whether iCT-SAD can retain the efficacy shown in UK studies when delivered in a different culture with minimal adaptation. It therefore sought to examine whether the UK findings could be replicated in a new sample, as well as evaluating the dissemination of the treatment to a different country. Hong Kong offers a clear cultural contrast to the UK, yet a sufficient English-speaking population to permit the treatment being implemented without translation at this initial stage. Online psychological therapy is rare in Hong Kong, though findings from a pilot case series of iCT-SAD demonstrated good initial evidence of feasibility and efficacy in this context (Thew et al., 2019). The present RCT aimed to examine whether iCT-SAD was superior to a waitlist control condition, and to benchmark the performance of iCT-SAD in Hong Kong against the results of UK studies.

## 2 Method

### 2.1 Design

The study was a two-arm, parallel-group randomized controlled superiority trial. Participants were randomly assigned to iCT-SAD or waitlist with an allocation ratio of 1:1. Randomisation was undertaken following a minimisation procedure by an independent research assistant in the UK, stratifying by baseline severity of social anxiety (Liebowitz Social Anxiety Scale – Self-Report score > 76, or ≤76, where 76 points represented the median score of iCT-SAD participants in the UK trial) and gender, which was an addition to the preregistered strategy. The principal assessment points were baseline, midtreatment/midwait (week 8), and posttreatment/postwait (week 15). Participants in the iCT group also completed a three-month follow-up assessment. The trial was prospectively registered (ISRCTN11357117) and approved by the Joint Chinese University of Hong Kong–New Territories East Cluster Clinical Research Ethics Committee (Ref: 2016.611-T), and the University of Oxford Tropical Research Ethics Committee (Ref: 531-17).

### 2.2 Participants

Inclusion criteria were: meets DSM-5 criteria for SAD (American Psychiatric Association, 2013); participant considers SAD to be their main problem; age 18–65 (inclusive); no current psychotropic mediation, or on a stable dose for at least two months without improvement, and willing to remain at this dose throughout trial; participant agrees not to start any other forms of treatment during the trial; participant is a Chinese resident of Hong Kong, with sufficient proficiency in English to understand the treatment content; internet access from home. Exclusion criteria were: current or past psychosis, bipolar disorder, or borderline personality disorder; active suicidality; dependence on alcohol or substances; currently receiving psychological treatment or having completed a course of CBT for social anxiety previously (defined as at least 5 sessions, and including an exposure component). These criteria, with the exception of geographical location, matched those used in the UK trial (Clark et al., submitted).

Recruitment was undertaken via advertisements in social and print media between November 2017 and April 2018. These directed potential participants to a brief online screening questionnaire comprised of the social anxiety items of the Psychiatric Diagnostic Screening Questionnaire (Zimmerman and Mattia, 2001) and some brief eligibility questions. If respondents' scores suggested they may meet criteria for SAD, they were invited to attend an assessment with one of four trained research assistants. The assessor and the first author then reviewed the assessment results against the eligibility criteria. Those eligible were invited to meet with one of the study therapists to discuss the treatment and study procedures prior to randomisation. All 44 participants (22 in each group) provided written informed consent to take part. In the Hong Kong healthcare system, payment for psychological therapy is made at the point of care. To align the present treatment with this model we therefore asked participants to pay a deposit of 2000HKD (approx. 200GBP), which is comparable to the initial fee associated with routine public sector face-to-face treatment. The deposit was waived in cases of financial difficulty, and all deposits were returned at the end of study participation.

The Anxiety and Related Disorders Interview Schedule for DSM-5 (ADIS; Brown and Barlow, 2014) was used to assess SAD diagnostic status, and the Structured Clinical Interview for DSM-5 (First et al., 2015) for all other comorbid conditions. Reliability of the independent assessors' diagnostic decisions was evaluated using a sample of six audio-recordings of initial ADIS assessments, included those with and without SAD. Full agreement between sets of three assessors per case was obtained for five of the six recordings, with the sixth being a participant with social anxiety in the subthreshold range. Fleiss' kappa was 0.77, which is considered ‘substantial agreement’ (Landis and Koch, 1977).

### 2.3 Treatment

iCT-SAD (Clark et al., submitted; Stott et al., 2013) is a therapist-guided modular online treatment based on the Clark and Wells (1995) cognitive model of social anxiety. It aims to replicate the content and procedures of the face-to-face cognitive therapy (CT-SAD) protocol (see Clark et al., 2006; Warnock-Parkes et al., 2020), which in the UK is a primary treatment recommendation for adults with SAD (NICE, 2013). The treatment and wait period were both 14 weeks in duration, after which the waitlist group began treatment. At the end of the 14-week treatment period, participants entered the booster phase of treatment, which lasted for a further three months. Participants retained access to the treatment website during this phase, and for at least a further 12 months.

In iCT-SAD, therapists communicate with their client via asynchronous messaging, SMS messaging, telephone calls, and occasional video calls via webcam. In the first two weeks of therapy, two telephone calls are scheduled per week, followed by one weekly call until the end of treatment. Up to three calls are scheduled at monthly intervals in the booster phase. Each call lasts approximately 15–20 min, and is used to review the client's questionnaires, review progress with treatment modules and behavioural experiments, and to plan for the coming week. The treatment protocol was identical to that of the previous UK trial. The treatment content was presented in English. The resources library in the programme also included Chinese versions of an attention training exercise and an exercise involving listening to a group conversation that therapists could recommend. Therapists' messages were written in English, and the telephone calls were conducted in English, Cantonese, or a combination of both as necessary.

### 2.4 Therapists

Treatment was delivered by three local clinical psychologists (AK, MLC, and CP), all of whom had previous experience of CBT interventions for anxiety, with a mean of 10.3 years post-qualification clinical experience. The iCT-SAD training programme and its evaluation is described in Thew et al. (2019). GT provided regular supervision, and received ‘supervision of supervision’ from JW, one of the trial therapists from the UK RCT, to ensure implementation was consistent with the UK study.

### 2.5 Measures

The primary outcome measure of the trial was the self-report version of the Liebowitz Social Anxiety Scale (LSAS; Baker et al., 2002). Secondary outcome measures were as follows.

#### 2.5.1 Social anxiety

We evaluated the proportion of participants no longer meeting SAD diagnostic criteria, using the Anxiety and Related Disorders Interview Schedule for DSM-5 (ADIS-5; Brown and Barlow, 2014) conducted by an independent assessor blind to treatment condition at the posttreatment/postwait assessment point. Social anxiety was also assessed using the Social Phobia Weekly Summary Scale (SPWSS; Clark et al., 2003; Oxford Centre for Anxiety Disorders and Trauma, 2019), Social Phobia Inventory (SPIN; Connor et al., 2000), Fear of Negative Evaluation Scale (FNE; Watson and Friend, 1969), Social Phobia Scale (SPS; Mattick and Clarke, 1998) and Social Interaction Anxiety Scale (SIAS; Mattick and Clarke, 1998).

#### 2.5.2 Social anxiety process measures

Psychological processes targeted by iCT-SAD were assessed using the Social Cognitions Questionnaire (SCQ: frequency and belief in negative thoughts), Social Behaviours Questionnaire (SBQ: safety behaviours), and Social Attitudes Questionnaire (SAQ: negative social anxiety related assumptions), described in Clark (2005). Social participation and satisfaction were assessed using Alden and Taylor's (2011) scales. This study also used the Generalised Learning Questionnaire (GLQ): an original, 5-item scale developed for the present study to examine longitudinal changes in generalised learning linked to social anxiety (see supplementary material).

#### 2.5.3 Depression, anxiety, and general functioning

Depression was assessed using the Patient Health Questionnaire (PHQ-9; Kroencke et al., 2001), anxiety using the Generalised Anxiety Disorder Questionnaire (GAD-7; Spitzer et al., 2006), and general functioning using the Work and Social Adjustment Scale (WSAS; Mundt et al., 2002).

#### 2.5.4 Non-specific therapy factors

After treatment week 2, patients and therapists completed the Working Alliance Inventory (Horvath and Greenberg, 1989; Tracey and Kokotovic, 1989). Patients also completed Borkovec and Nau's (1972) treatment credibility scale.

### 2.6 Sample size

An a priori sample size calculation for the between-group comparison of LSAS scores from baseline to posttreatment was computed. As the controlled effect size (*d*_Cohen_) for the iCT-SAD group reported in the UK RCT (Clark et al., submitted) was over 2, a conservative estimated effect size of half that value (*d* = 1.1) was used given the different population and cultural setting of this study. This calculation (using β = 0.9 and α = 0.05) indicated a required sample of 13 participants per group. Given the further planned analyses of secondary measures and accounting for attrition, 20 participants per group were sought.

### 2.7 Response, remission, and deterioration criteria

Response to treatment and remission from SAD were calculated as per Stott et al.' (2013). Response was defined as an improvement on the LSAS between pretreatment and posttreatment greater than 31% (Bandelow et al., 2006). Remission was defined as a drop of at least 12 LSAS points combined with a posttreatment score of 38 or less (Clark et al., 2006). A lower remission threshold of 30 points was also examined given its use in some studies (Johansson et al., 2017; Leichsenring et al., 2014). Reliable deterioration, considered an adverse event (Rozental et al., 2014), was defined as an increase on the LSAS of at least 12 points. For comparability with UK samples, we also computed the Improving Access to Psychological Therapies (IAPT) programme recovery and reliable improvement rates that simultaneously consider change on the SPIN and PHQ-9 (National Collaborating Centre for Mental Health, 2021).

### 2.8 Analysis

Analyses were performed in R version 3.4.3 (R Core Team, 2017) using the ‘tidyverse’ (Wickham, 2017), ‘nlme’ (Pinheiro et al., 2018), ‘jmv’ (Selker et al., 2018) and ‘psych’ (Revelle, 2018) packages. All analyses were performed on the intention to treat sample unless specified, using an alpha level of *p* = 0.05. Preliminary checks were performed to examine the distribution of the data and check for outliers. No transformations or exclusions were indicated. Descriptive statistics are reported for data regarding participant demographics, iCT participants' use of the site, and therapists' activity.

Linear mixed effect models were used for the analysis of continuous variables over time, given their ability to include all available data from all randomized participants, to account for repeated measures and data missing at random. Time (midwait/midtreatment, and postwait/posttreatment), condition (iCT, waitlist), and the time-by-condition interaction (to allow estimation of treatment effect at each timepoint) were specified as categorical fixed factors, with the stratification variables of baseline LSAS score and gender as fixed covariates, and participant as a random effect to account for between-person variation. For the analysis of secondary outcome measures, the baseline score of the measure being analysed was also included as a fixed covariate. All models used restricted maximum likelihood estimation. Q-Q plots indicated that the normality of residuals assumption was met for all models. Results consistent with the hypothesis of superior treatment effects in the iCT group compared to waitlist would therefore be indicated by significant adjusted group differences at a given timepoint, with greater mean change in iCT. Between-group effect sizes (*d*_Cohen_) were calculated by dividing the adjusted group difference by the pooled standard deviation at the relevant timepoint. Within-group effect sizes were calculated from linear mixed effects models that incorporated the baseline score as a timepoint rather than as a covariate, to obtain within-group adjusted means in relation to baseline. These models used an unstructured covariance matrix. 95% confidence intervals for *d*_Cohen_ were calculated by dividing the upper and lower limits of the adjusted group difference by the pooled standard deviation.

Categorical outcomes were analysed using Chi-squared tests. Benchmarking was performed through descriptive comparisons of the present results in relation to UK studies of iCT-SAD (Clark et al., submitted; Stott et al., 2013). Lastly, exploratory analyses were performed to examine candidate mediators of the relationship between randomisation (treatment condition) and post-treatment LSAS scores, following the procedure described by Freeman et al. (2017). This analysis is similar to the Baron and Kenny (1986) approach but uses linear mixed effects models at each step to account for the nested data structure. The candidate mediators were self-focused attention (the general self-focused attention item of the SPWSS), negative social cognitions (SCQ), depressed mood (PHQ), safety behaviours (SBQ) and rumination (the rumination item of the SPWSS). Midwait/midtreatment scores were used as the mediator, and posttreatment LSAS scores as the outcome. Models reversing the mediator and outcome variable but maintaining the temporal lag were also performed. All models included the stratification variables and baseline mediator scores as covariates.

## 3 Results

### 3.1 Participant flow and demographics

**Fig. 1:**
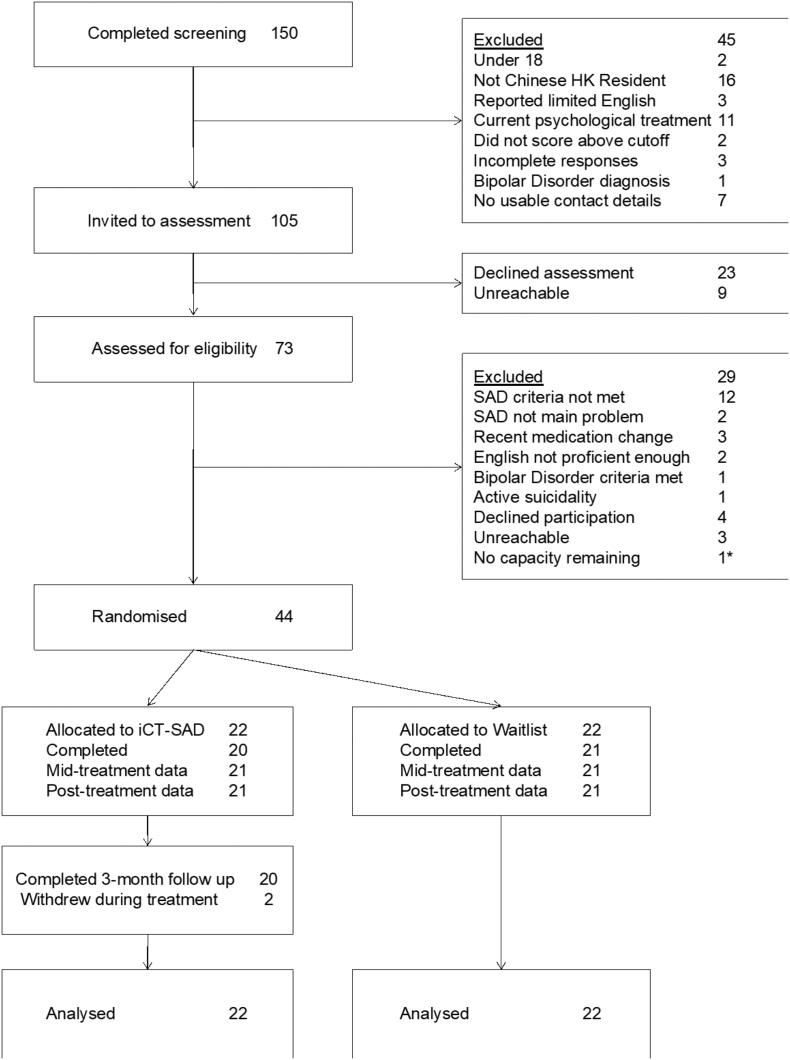
Participant flow through the trial. *One person, who was assessed at the end of the recruitment period, was eligible but could not be randomized due to a lack of capacity among the trial therapists. Alternative treatment arrangements were made.

The flow of recruitment and participation is shown in Fig. 1.

Of the 73 potential participants who were assessed, 44 were eligible for the trial, gave informed consent, and were therefore randomized. Demographic and clinical characteristics of the two groups are shown in Table 1. There were no significant differences between groups on the stratification variables of baseline LSAS score: *t*(42) = −0.523, *p* = 0.604, or gender: *χ*^2^(1) = 0.109, *p* = 0.741, indicating randomisation was successful. Two participants in the iCT condition withdrew during the study; one prior to starting treatment, and one during Week Two. Both provided at least some subsequent data. In the waitlist condition, one participant could not be contacted following randomisation. Overall, complete data was provided for 21 participants (95%) in each group.

**Table 1:** Participant demographic and clinical characteristics.

	iCT (n = 22)	Waitlist (n = 22)	Total (n = 44)
% Female	68	73	70
Mean age (SD)	34.5 (10.4)	31.7 (8.4)	33.1 (9.4)
Age range	20–54	20–48	20–54
Marital status n (%)			
Single/living alone	16 (73)	13 (59)	29 (66)
Married/living together	5 (23)	9 (41)	14 (32)
Divorced/widowed/separated	0	0	0
Other	1 (5)	0	1 (2)
Highest educational qualification n (%)			
No formal qualification/Primary school	0	0	0
Secondary school	1 (5)	0	1 (2)
Associate degree/high diploma	1 (5)	1 (5)	2 (5)
Bachelor's degree	13 (59)	15 (68)	28 (64)
Master's degree	6 (27)	6 (27)	12 (27)
Doctoral degree	1 (5)	0	1 (2)
Other	0	0	0
Employment status n (%)			
Unemployed	1 (5)	1 (5)	2 (5)
Full time work	14 (64)	14 (64)	28 (64)
Part time work	0	1 (5)	1 (2)
Student	5 (23)	3 (14)	8 (18)
Homemaker	1 (5)	1 (5)	2 (5)
Retired	0	1 (5)	1 (2)
Sick leave	1 (5)	1 (5)	2 (2)
Mean age at SAD onset in years (SD)	16.3 (7.1)	16.9 (9.6)	16.6 (8.3)
Mean duration of SAD at assessment in years (SD)	18.7 (12.9)	14.8 (10.6)	16.8 (11.8)
% ‘Performance only’ SAD subtype	0	0	0
Current psychotropic medication (%)	3 (14)	1 (5)	4 (9)
Previous psychological treatment (%)	9 (41)	6 (27)	15 (34)
Comorbidity (current) n (%)			
Major depressive episode	2 (9)	2 (9)	4 (9)
Generalised anxiety disorder	4 (18)	4 (18)	8 (18)
Panic disorder	1 (5)	0	1 (2)
Specific phobia	2 (9)	0	2 (5)
Somatic symptom disorder	0	1 (5)	1 (2)
Illness anxiety disorder	1 (5)	0	1 (2)
Alcohol use disorder (mild)	1 (5)	0	1 (2)
Body dysmorphic disorder	0	1 (5)	1 (2)
Avoidant personality disorder	12 (55)	8 (36)	20 (45)
Obsessive compulsive personality disorder	1 (5)	2 (9)	3 (7)
Paranoid personality disorder	0	1 (5)	1 (2)
Any current comorbidity	14 (64)	12 (55)	26 (59)

### 3.2 Use of the iCT Programme and therapeutic Alliance

The participants allocated to iCT who completed treatment (n = 20) spent an average of 2611 min (43.5 h) on the website across the 14-week programme (SD = 948). This was comparable to the UK trial (Clark et al., submitted). All participants completed the ‘core’ treatment modules and at least six additional ‘optional’ modules tailored to their specific concerns or difficulties. They completed a mean of 15.2 (SD = 10.5) behavioural experiments. They sent a mean of 9.2 messages (SD = 14.4) to their therapist through the website, though it is noted that most participants preferred to message their therapist via WhatsApp. All participants who completed treatment used the webchat facility for the ‘self-focused attention and safety behaviours experiment’ and video feedback module, where they recorded and watched videos of themselves engaging in social interactions. The mean participant-rated alliance score was 67.8 (SD = 9.8), which was similar to the therapists' mean rating of 66.4 (SD = 4.3). The mean participant-rated treatment credibility score was 7.4 out of 10 (SD = 1.3).

### 3.3 Therapist activity

Across the 14-week intervention, therapists made a mean of 15.3 phone calls (SD = 1.1) to the iCT participants, with a mean duration of 23.1 min (SD = 6.6), and thus a mean total time of 352 min per participant (SD = 101). They completed a mean of 1.5 webchats (SD = 0.5), with a total duration of 95.1 min (SD = 13.9), nearly all of which was used to complete the self-focused attention and safety behaviours experiment in Week Two. Overall, the mean time spent in direct communication with participants during iCT-SAD was 7.5 h (SD = 1.85), which is substantially less than the 19 h required to deliver a course of face-to-face CT (Clark et al., 2006). Therapists sent a mean of 28.6 messages (SD = 11.8) through the website, and a mean of 89.3 text messages (SD = 67.7) to participants' mobile phones. The mean time spent reviewing participants' work on the site and sending messages was 208 min (3.5 h) (SD = 82). It therefore seems that iCT-SAD represented a saving in therapists' contact time of around 60% compared to the face-to-face protocol.

### 3.4 Primary outcomes

**Fig. 2:**
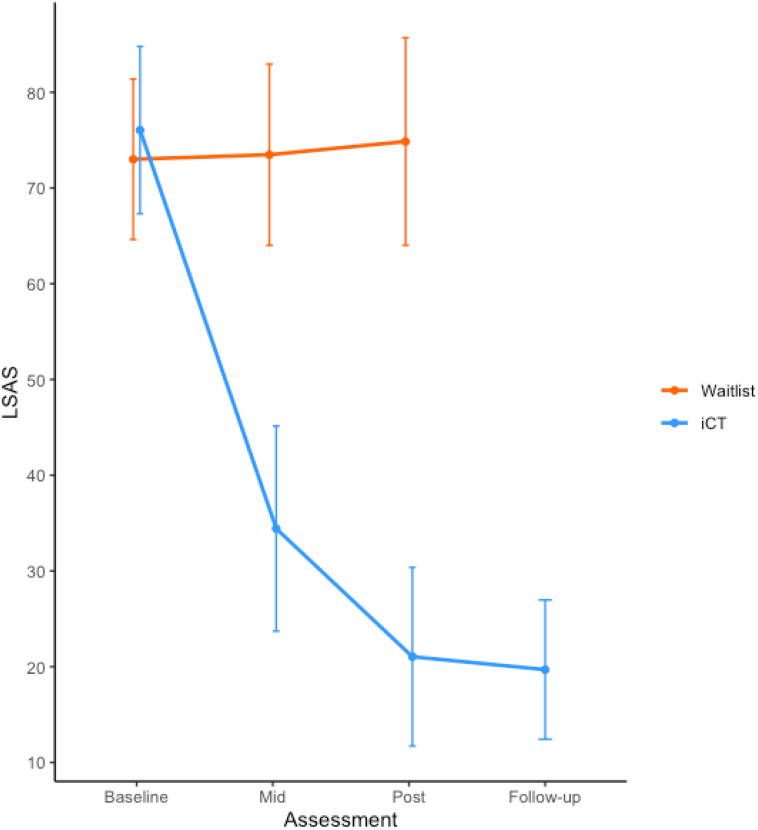
Mean scores on Liebowitz Social Anxiety Scale (self-report version; LSAS) at each assessment point. Error bars represent 95%CI.

Mean LSAS scores at the principal timepoints are shown in Fig. 2. Results indicated that the difference between groups in their change over time was significant at the mid and post timepoints, with iCT showing the superior treatment effect. The between-group effect size at posttreatment was 2.41 (*d*_Cohen_), which is considered large. Treatment gains were maintained at 3-month follow-up. Table 2 reports the unadjusted means and standard deviations, adjusted group differences, and effect sizes for all continuous outcomes.

**Table 2:** Unadjusted means, standard deviations, adjusted differences, and effect sizes of the primary and secondary outcome measures for the intention to treat sample.

Measure and condition	Unadjusted mean (*SD*)				Adjusted difference (*SE*) [95%CI], *p* value		Effect size *d*_Cohen_ [95%CI]			
	Pre	Mid	Post	3-month FU	Mid	Post	Between-group at mid	Between-group at post	Within-group pre-post	Within-group pre-FU
LSAS										
iCT	76.05 (19.72)	34.43 (23.53)	21.05 (21.03)	19.70 (15.54)	39.48 (5.50) [28.36, 50.60], <0.001	55.36 (5.45) [44.32, 66.39], <0.001	1.73 [1.25, 2.22]	2.41 [1.93, 2.89]	2.64 [2.27, 3.00]	3.03 [2.55, 3.52]
Wait	73.00 (18.90)	73.48 (20.80)	74.86 (23.81)						0.06 [−0.29, 0.41]	

SPWSS										
iCT	27.05 (4.99)	16.38 (9.15)	13.00 (6.89)	12.35 (7.98)	10.18 (2.02) [6.08, 14.27], <0.001	14.16 (2.01) [10.10, 18.23], <0.001	1.12 [0.67, 1.57]	1.87 [1.34, 2.41]	2.28 [1.84, 2.72]	2.17 [1.73, 2.61]
Wait	27.55 (7.29)	27.00 (8.58)	27.43 (7.86)						0.02 [−0.34, 0.37]	

SPIN										
iCT	42.09 (10.54)	21.38 (12.12)	15.67 (11.38)	13.45 (10.23)	17.81 (3.04) [11.65, 23.97], <0.001	24.69 (3.04) [18.53, 30.85], <0.001	1.41 [0.92, 1.90]	2.04 [1.53, 2.55]	2.38 [1.97, 2.79]	2.67 [2.16, 3.18]
Wait	40.36 (12.84)	38.90 (12.55)	38.90 (12.18)						0.13 [−0.23, 0.49]	

FNE										
iCT	26.41 (2.63)	22.43 (5.90)	20.90 (6.84)	18.25 (6.44)	4.58 (1.60) [1.35, 7.81], 0.007	5.82 (1.60) [2.58, 9.06], 0.001	0.79 [0.23, 1.35]	0.95 [0.42, 1.47]	1.09 [0.69, 1.48]	1.67 [1.16, 2.18]
Wait	23.68 (5.06)	25.10 (5.40)	24.29 (5.05)						0.08 [−0.32, 0.48]	

SPS										
iCT	27.18 (14.32)	14.52 (12.03)	9.57 (7.83)	6.95 (8.76)	12.67 (2.99) [6.62, 18.72], <0.001	17.27 (2.99) [11.22, 23.32], <0.001	0.92 [0.48, 1.37]	1.45 [0.94, 1.96]	1.50 [1.13, 1.88]	1.64 [1.23, 2.04]
Wait	22.50 (13.53)	25.19 (14.61)	23.86 (14.46)						0.10 [−0.21, 0.41]	

SIAS										
iCT	44.50 (12.81)	29.86 (14.15)	25.67 (10.71)	20.70 (8.49)	14.29 (3.10) [8.02, 20.55], <0.001	19.98 (3.10) [13.70, 26.25], <0.001	0.98 [0.55, 1.41]	1.67 [1.15, 2.20]	1.57 [1.24, 1.91]	2.10 [1.67, 2.52]
Wait	48.91 (10.75)	47.43 (14.35)	47.57 (12.56)						0.10 [−0.24, 0.44]	

SCQ-frequency										
iCT	3.11 (0.50)	1.88 (0.64)	1.61 (0.69)	1.48 (0.53)	0.99 (0.21) [0.57, 1.41], <0.001	1.22 (0.21) [0.81, 1.64], <0.001	1.29 [0.75, 1.85]	1.68 [1.11, 2.25]	2.44 [2.05, 2.83]	3.03 [2.55, 3.51]
Wait	2.71 (0.49)	2.59 (0.83)	2.47 (0.74)						0.39 [0.01, 0.77]	

SCQ-belief										
iCT	59.38 (14.89)	25.78 (19.43)	16.36 (17.46)	13.16 (14.99)	34.51 (6.66) [21.03, 47.99], <0.001	40.22 (6.64) [26.78, 53.65], <0.001	1.57 [0.96, 2.19]	1.82 [1.21, 2.42]	2.59 [2.17, 3.01]	2.96 [2.49, 3.43]
Wait	42.71 (13.82)	48.57 (23.20)	42.75 (25.25)						<0.01 [−0.34, 0.35]	

SBQ										
iCT	36.68 (9.31)	24.62 (7.28)	20.90 (8.43)	20.80 (8.48)	10.13 (2.33) [5.41, 14.85], <0.001	14.15 (2.33) [9.43, 18.86], <0.001	1.20 [0.64, 1.75]	1.56 [1.04, 2.08]	1.75 [1.35, 2.15]	1.73 [1.17, 2.29]
Wait	36.50 (8.22)	35.10 (9.14)	34.57 (9.22)						0.22 [−0.19, 0.63]	

SAQ										
iCT	201.68 (27.53)	160.00 (39.45)	148.14 (32.30)	142.75 (33.56)	41.34 (7.53) [26.09, 56.60], <0.001	53.04 (7.54) [37.79, 68.30], <0.001	1.25 [0.79, 1.71]	1.79 [1.28, 2.31]	1.79 [1.48, 2.10]	1.92 [1.53, 2.30]
Wait	185.77 (27.41)	189.76 (23.26)	186.43 (24.96)						0.03 [−0.32, 0.38]	

Participation										
iCT	48.27 (13.53)	53.38 (15.31)	54.33 (10.59)	58.10 (14.55)	−8.96 (3.31) [−15.67, −2.25], 0.010	−10.16 (3.32) [−16.88, −3.44], 0.004	0.67 [0.17, 1.18]	0.97 [0.33, 1.62]	0.48 [0.05, 0.91]	0.65 [0.23, 1.08]
Wait	40.82 (11.43)	40.24 (10.08)	40.33 (9.77)						0.03 [−0.46, 0.52]	

Satisfaction										
iCT	21.27 (7.21)	22.76 (6.66)	24.38 (6.52)	24.75 (5.78)	−2.95 (1.25) [−5.47, −0.42], 0.024	−4.75 (1.25) [−7.28, −2.23], 0.001	0.49 [0.07, 0.92]	0.81 [0.38, 1.25]	0.48 [0.20, 0.76]	0.55 [0.27, 0.82]
Wait	17.95 (6.45)	17.71 (4.86)	17.90 (4.74)						0.01 [−0.33, 0.35]	

PHQ										
iCT	9.18 (4.85)	5.14 (5.10)	3.59 (4.39)	3.80 (4.21)	3.98 (1.17) [1.61, 6.34], 0.002	4.78 (1.16) [2.43, 7.14], <0.001	0.69 [0.28, 1.09]	0.86 [0.44, 1.28]	1.18 [0.84, 1.52]	1.19 [0.86, 1.51]
Wait	10.41 (6.01)	9.71 (6.16)	8.90 (6.38)						0.19 [−0.07, 0.44]	

GAD										
iCT	8.41 (5.00)	4.48 (4.74)	3.59 (3.42)	3.70 (3.80)	3.55 (1.22) [1.08, 6.02], 0.006	4.82 (1.21) [2.36, 7.28], <0.001	0.70 [0.21, 1.19]	0.99 [0.48, 1.50]	1.10 [0.70, 1.49]	1.04 [0.68, 1.41]
Wait	8.45 (4.54)	7.86 (5.11)	8.14 (5.83)						0.03 [−0.30, 0.36]	

WSAS										
iCT	14.36 (8.44)	8.33 (7.44)	5.73 (5.78)	6.00 (8.03)	6.75 (1.76) [3.19, 10.31], 0.001	9.09 (1.75) [5.54, 12.63], <0.001	0.84 [0.40, 1.28]	1.36 [0.83, 1.90]	1.17 [0.81, 1.52]	1.04 [0.72, 1.36]
Wait	15.52 (7.24)	15.50 (8.26)	15.17 (7.18)						0.04 [−0.32, 0.40]	

GLQ										
iCT	14.82 (3.16)	18.43 (3.53)	18.50 (4.06)	19.45 (4.25)	−2.79 (1.24) [−5.30, −0.29], 0.030	−3.31 (1.23) [−5.81, −0.82], 0.011	0.74 [0.08, 1.41]	0.78 [0.19, 1.36]	0.99 [0.46, 1.52]	1.21 [0.71, 1.71]
Wait	15.41 (4.56)	15.52 (3.82)	15.33 (4.29)						0.01 [−0.43, 0.44]	

*Note*. In the iCT group, 22 participants provided complete data at baseline, 21 at midtreatment and posttreatment, and 20 at 3-month follow-up. In the wait group, 22 participants provided data at baseline, and 21 at midtreatment and posttreatment. LSAS = Liebowitz Social Anxiety Scale; SPWSS = Social Phobia Weekly Summary Scale; SPIN = Social Phobia Inventory; FNE = Fear of Negative Evaluation Scale; SPS = Social Phobia Scale; SIAS = Social Interaction Anxiety Scale; SCQ = Social Cognitions Questionnaire (mean scores); SBQ = Social Behaviour Questionnaire; SAQ = Social Attitudes Questionnaire; PHQ = Patient Health Questionnaire; GAD = Generalised Anxiety Disorder Questionnaire; WSAS = Work and Social Adjustment Scale; GLQ = Generalised Learning Questionnaire.

Table 3 shows the rates of response and remission for the various criteria used (see Method section). For all indices, improvement was significantly greater in the iCT group than the wait group. Three participants showed reliable deterioration across the baseline-to-posttreatment interval, all of whom were in the waitlist group.

**Table 3:** Response and Remission rates for the different criteria examined.

	iCT	Wait	χ^2^	*p*
Response to treatment	95% (21/22)	5% (1/22)	36.36	<0.001
Remission	86% (19/22)	5% (1/22)	29.70	<0.001
Remission (30-point cutoff)	77% (17/22)	5% (1/22)	24.07	<0.001
IAPT reliable improvement	82% (18/22)	36% (8/22)^b^	9.40	0.002
IAPT reliable recovery	68% (15/22)	5% (1/22)	19.25	<0.001
Loss of SAD diagnosis (ADIS-5)^a^	73% (16/22)	9% (2/22)	18.43	<0.001

a One waitlist and three iCT participants did not complete this interview, so loss of diagnosis could not be demonstrated. The posttreatment LSAS scores for the iCT participants were 6, 9, and 55, suggesting two may have no longer met diagnostic criteria at interview.

b Of these eight participants, three showed reliable improvement on the PHQ only, and five on the SPIN only.

### 3.5 Secondary outcomes

Significant group differences at postwait/posttreatment in favour of iCT were found for all secondary measures, with between-group effect sizes ranging between 0.78 and 2.04, and treatment gains maintained at 3-month follow-up (see Table 2). Of the four participants who were taking psychotropic medication at baseline, one iCT participant reported having stopped. No other participants changed medication status.

### 3.6 Benchmarking against UK efficacy

Table 4 shows a comparison of the present findings with the initial UK study (Stott et al., 2013). Participants in Hong Kong showed at least as much improvement as those in the UK. The Hong Kong effect size is also similar to that observed in the more recent UK clinical trial (Clark et al., submitted).

**Table 4:** Comparison of Results with UK Study (Intention-to-Treat).

	Stott et al. (2013) development case series	Present RCT (iCT-SAD group)
Location	UK	Hong Kong
N	11	22
Mean LSAS at baseline (SD)	80.0 (24.6)	76.1 (19.7)
Mean LSAS at post (SD)	39.8 (30.1)	21.1 (21.0)
Within-group *d*_Cohen_ (pre-post)	1.64	2.64
Response rate (%)	82	95
Remission rate (%)	64	86

*Notes*. The criteria to define response and remission were the same across studies and relate to the posttreatment assessment.

### 3.7 Mediation of clinical improvement

Results of the mediation models are shown in Table 5. Midwait/midtreatment scores on self-focused attention, negative social cognitions, depressed mood, safety behaviours, and rumination were all found to significantly mediate the relationship between randomisation (treatment condition) and postwait/posttreatment scores on the LSAS, with negative social cognitions (SCQ belief subscale) and safety behaviours showing the strongest effect. Models reversing the mediator and outcome variables also showed significant mediation for all candidate mediators except the belief subscale for negative social cognitions, with generally larger percent mediation values. This may indicate a cyclical relationship between changes in process variables and changes in symptoms.

**Table 5:** Mediation of postwait/posttreatment scores.

Mediator (at mid)	Total effect		Direct effect		Indirect effect		% Mediated
	Adjusted difference [95%CI]	*p*	Adjusted difference [95%CI]	*p*	Adjusted difference [95%CI]	*p*	
Outcome = LSAS at post							
SFA	−55.28 [−66.30, −44.26]	<0.001	−49.09 [−60.47, −37.72]	<0.001	−7.21 [−13.36, −1.06]	0.022	13
SCQ-f	−61.30 [−72.61, −49.99]	<0.001	−52.53 [−65.73, −39.33]	<0.001	−8.82 [−16.68, −0.95]	0.028	14
SCQ-b	−59.43 [−72.22, −46.64]	<0.001	−47.77 [−63.40, −32.14]	<0.001	−12.16 [−22.38, −1.95]	0.020	20
PHQ	−55.09 [−66.29, −43.88]	<0.001	−48.09 [−59.83, −36.34]	<0.001	−7.99 [−14.95, −1.02]	0.025	14
SBQ	−55.38 [−66.46, −44.29]	<0.001	−44.85 [−58.09, −31.61]	<0.001	−10.84[−19.69, −2.00]	0.016	20
Rumination	−54.43 [−65.17, −43.68]	<0.001	−48.59 [−59.56, −37.63]	<0.001	−6.91 [−12.72, −1.10]	0.020	13


‘Reversed’ models: mediator = LSAS at mid							
Outcome (at post)	Total effect		Direct effect		Indirect effect		% Mediated
	Adjusted difference [95%CI]	*p*	Adjusted difference [95%CI]	*p*	Adjusted difference [95%CI]	*p*	
SFA	−2.23 [−3.18, −1.27]	<0.001	−0.86 [−2.18, 0.46]	0.186	−1.41 [−2.41, −0.42]	0.005	64
SCQ-f	−1.22 [−1.64, −0.81]	<0.001	−0.66 [−1.20, −0.13]	0.012	−0.63 [−1.06, −0.20]	0.004	52
SCQ-b	−40.22 [−53.65, −26.78]	<0.001	−31.42 [−50.19, −12.64]	0.001	−10.65 [−24.07, 2.77]	0.120	–
PHQ	−4.78 [−7.13, −2.43]	<0.001	−1.08 [−4.16, 2.00]	0.476	−3.90 [−6.31, −1.49]	0.002	82
SBQ	−14.15 [−18.86, −9.43]	<0.001	−9.13 [−15.32, −2.93]	0.003	−5.96 [−10.59, −1.32]	0.012	42
Rumination	−2.81 [−3.83, −1.80]	<0.001	−1.32 [−2.67, 0.03]	0.047	−1.53 [−2.53, −0.53]	0.003	54

*Notes*. *N* = 44. LSAS = Liebowitz Social Anxiety Scale (Self-Report); SFA = Self-focused attention (SPWSS); SCQ-f = Social Cognitions Questionnaire (frequency subscale); SCQ-b = Social Cognitions Questionnaire (belief subscale); PHQ = Patient Health Questionnaire; SBQ = Social Behaviour Questionnaire; Rumination = SPWSS rumination item.

## 4 Discussion

The main aim of this study was to examine whether iCT-SAD was superior to a waitlist control condition when delivered in Hong Kong, a cultural context different to where the treatment was developed. Results indicated that the treatment group showed significantly greater reductions in social anxiety compared to waitlist, with between- and within-group effect sizes (*d*_Cohen_) of 2.41 and 2.64 respectively. 95% of the treatment group were classified as treatment responders, and 86% as remitted from social anxiety at the posttreatment assessment. As the treatment was completed by 20 out of 22 participants (91%), the present findings support those of previous work (Thew et al., 2019) in suggesting that the treatment was feasible to implement and acceptable to participants in this setting. The present results were comparable to UK studies of iCT-SAD, showing an effect size that compares favourably with those observed in other ICBT interventions for SAD (see Kampmann et al., 2016). It is possible that the high efficacy of iCT-SAD may be related to treatment components (such as the in-programme video conference facility to support behavioural experiments and video feedback, and the trauma memory work), which are less common in other internet-delivered SAD interventions. The use of a deposit system in the present study may have enhanced motivation and compliance, though similar results have been obtained in UK studies, where the deposit scheme was not used. iCT-SAD may therefore represent one of the most efficacious internet interventions for social anxiety and was transported to Hong Kong without substantial loss of efficacy. This is consistent with the results of other studies implementing online treatments in new cultural contexts (Gallego et al., 2011; Jakobsen et al., 2017; Kishimoto et al., 2016; Tulbure et al., 2015), and the results of a pilot case series (Thew et al., 2019). This is one of the first trials of an internet intervention in Hong Kong, and demonstrates the potential of this treatment modality in this setting, given the positive findings regarding feasibility, acceptability to participants, completion rates, and clinical outcomes. The present amount of therapist contact time per patient was comparable to UK studies and represents a time saving of around 60% compared to an equivalent face-to-face treatment.

This is the first study of its type to examine an untranslated and largely unadapted treatment. The outcomes suggest that the influence of culture in this particular setting (English-speaking Chinese residents in Hong Kong) was not such that patients' engagement, understanding, or progress within the treatment was significantly hindered. This of course does not mean that cultural factors had no influence on treatment. It is possible that the therapists' tailoring of treatment content in terms of module selection and suggesting individualised behavioural experiments may have been sufficient to address any aspects of SAD that were more culture specific. Adaptations to the programme may still confer additional benefits, but among this sample there did not appear to be any major cultural barriers to understanding and implementing the treatment procedures. The present findings suggest that where possible, testing treatments in their largely unadapted form may be an efficient and practical first step, as it helps researchers to more clearly understand what adaptations may or may not be required.

Exploratory mediation models suggested that the observed posttreatment effect on the LSAS was mediated by prior scores on a range of process variables, namely self-focused attention, negative social cognitions (SCQ frequency and belief), depressed mood, safety behaviours, and rumination. Significant reverse mediation was also shown for all but one (SCQ belief) of these variables, and strong mediation effects were observed in this direction, suggesting a cyclical relationship. These findings are consistent with those of a recent study examining processes of change in CT-SAD delivered face-to-face (Thew et al., 2020). Although baseline scores on the mediator and outcome variables were accounted for in these models, it is possible that the strength of the present mediation effects may be underestimated given that much of the overall change on these variables occurred prior to the midpoint assessment. Further examination of mediation effects at a week-to-week level is recommended.

### 4.1 Limitations

Possible limitations of the study are a low proportion of male participants and recruitment from the community, which has benefits in that people could self-refer, but may limit generalisability to clinic samples. Similar results have been obtained with recruitment from clinical services (Thew et al., 2019), but further work in clinic settings is recommended (see Thew, 2020). The requirement of English proficiency may have resulted in younger, more educated participants being overrepresented in the sample. Translation of the programme into Chinese may permit wider recruitment. The baseline internal consistency of the SPWSS was low compared to other measures, and for the GLQ this did not reach an acceptable level, so results from these measures should be interpreted with caution. Lastly, the use of a waitlist control condition means we were not able to determine the extent to which the improvements associated with iCT-SAD were due to specific versus non-specific therapy factors. A wait list control was deemed appropriate given this was the first trial of iCT-SAD in Hong Kong, and that waiting periods for psychological therapy are common in its public hospitals and clinics. However, using active control conditions in future studies would allow more detailed research questions to be addressed.

### 4.2 Conclusion

The results indicate that the high efficacy of iCT-SAD observed in UK studies was maintained when the treatment was transported and implemented by local therapists in Hong Kong. This study adds to the body of evidence that SAD can be treated successfully using internet cognitive-behavioural interventions, and that the transportation of these from one culture to another is possible to achieve without substantial loss of efficacy. Internet interventions such as iCT-SAD may therefore provide a promising route to increase the international dissemination of evidence-based psychological therapies.

## Declaration of competing interest

The authors have no conflicts to declare.

## Appendix A Supplementary data

Supplementary material Image 1

## References

[bb0005] Alden L.E., Taylor C.T. (2011). Relational treatment strategies increase social approach behaviors in patients with generalized social anxiety disorder. J.Anxiety Disord..

[bb0010] American Psychiatric Association (2013).

[bb0015] Baker S.L., Heinrichs N., Kim H.-J., Hofmann S.G. (2002). The Liebowitz social anxiety scale as a self-report instrument: a preliminary psychometric analysis. Behav. Res. Ther..

[bb0020] Bandelow B., Baldwin D.S., Dolberg O.T., Andersen H.F., Stein D.J. (2006). What is the threshold for symptomatic response and remission for major depressive disorder, panic disorder, social anxiety disorder, and generalized anxiety disorder?. J.Clin.Psychiatry.

[bb0025] Baron R.M., Kenny D.A. (1986). The moderator–mediator variable distinction in social psychological research: conceptual, strategic, and statistical considerations. J. Pers. Soc. Psychol..

[bb0030] Bernal G., Jiménez-Chafey M.I., Domenech Rodríguez M.M. (2009). Cultural adaptation of treatments: a resource for considering culture in evidence-based practice. Prof. Psychol. Res. Pract..

[bb0035] Borkovec T.D., Nau S.D. (1972). Credibility of analogue therapy rationales. J. Behav. Ther. Exp. Psychiatry.

[bb0040] Brown T.A., Barlow D.H. (2014).

[bb0045] Clark D.M. (2005).

[bb0065] Clark, D. M., Wild, J., Warnock-Parkes, E., Stott, R., Grey, N., Thew, G., Ehlers, A., (submitted). More than doubling the clinical benefit of each hour of therapist time: A randomized controlled trial of internet cognitive therapy for social anxiety disorder. 10.1017/S0033291722002008 PMC10476054 35835726

[bb0050] Clark D.M., Ehlers A., Hackmann A., McManus F., Fennell M., Grey N., Wild J. (2006). Cognitive therapy versus exposure and applied relaxation in social phobia: a randomized controlled trial. J. Consult. Clin. Psychol..

[bb0055] Clark D.M., Ehlers A., McManus F., Hackmann A., Fennell M., Campbell H., Louis B. (2003). Cognitive therapy versus fluoxetine in generalized social phobia: a randomized placebo-controlled trial. J. Consult. Clin. Psychol..

[bb0060] Clark D.M., Wells A., Heimberg R.G., Liebowitz M., Hope D.A., Schneier F. (1995). Social Phobia: Diagnosis, Assessment, And Treatment.

[bb0070] Connor K.M., Davidson J.R., Churchill L.E., Sherwood A., Weisler R.H., Foa E. (2000). Psychometric properties of the Social Phobia Inventory (SPIN) new self-rating scale. Br. J. Psychiatry.

[bb0075] First M.B., Williams J.B.W., Karg R.S., Spitzer R.L. (2015).

[bb0080] Freeman D., Sheaves B., Goodwin G.M., Yu L.-M., Nickless A., Harrison P.J., Wadekar V. (2017). The effects of improving sleep on mental health (OASIS): a randomised controlled trial with mediation analysis. Lancet Psychiatry.

[bb0085] Gallego M.J., Emmelkamp P.M.G., van der Kooij M., Mees H. (2011). The effects of a Dutch version of an internet-based treatment program for fear of public speaking: a controlled study. Int. J. Clin. Health Psychol..

[bb0090] Horvath A.O., Greenberg L.S. (1989). Development and validation of the Working Alliance Inventory. J. Couns. Psychol..

[bb0095] Hwang W.-C. (2009). The Formative Method for Adapting Psychotherapy (FMAP): a community-based developmental approach to culturally adapting therapy. Prof. Psychol. Res. Pract..

[bb0100] Jakobsen H., Andersson G., Havik O.E., Nordgreen T. (2017). Guided internet-based cognitive behavioral therapy for mild and moderate depression: a benchmarking study. Internet Interv..

[bb0105] Johansson R., Hesslow T., Ljótsson B., Jansson A., Jonsson L., Färdig S., Lilliengren P. (2017). Internet-based affect-focused psychodynamic therapy for social anxiety disorder: a randomized controlled trial with 2-year follow-up. Psychotherapy.

[bb0110] Kampmann I.L., Emmelkamp P.M., Morina N. (2016). Meta-analysis of technology-assisted interventions for social anxiety disorder. J.Anxiety Disord..

[bb0115] Kishimoto T., Krieger T., Berger T., Qian M., Chen H., Yang Y. (2016). Internet-based cognitive behavioral therapy for social anxiety with and without guidance compared to a wait list in China: a propensity score study. Psychother. Psychosom..

[bb0120] Kroencke K., Spitzer R., Williams J. (2001). The PHQ-9: validity of a brief depression severity measure. J. Gen. Intern. Med..

[bb0125] Landis J.R., Koch G.G. (1977). The measurement of observer agreement for categorical data. Biometrics.

[bb0130] Leichsenring F., Salzer S., Beutel M.E., Herpertz S., Hiller W., Hoyer J., Poehlmann K. (2014). Psychodynamic therapy and cognitive-behavioral therapy in social anxiety disorder: a multicenter randomized controlled trial. Am. J. Psychiatry.

[bb0135] Mattick R.P., Clarke J.C. (1998). Development and validation of measures of social phobia scrutiny fear and social interaction anxiety. Behav. Res. Ther..

[bb0140] Mundt J.C., Marks I.M., Shear M.K., Greist J.M. (2002). The Work and Social Adjustment Scale: a simple measure of impairment in functioning. Br. J. Psychiatry.

[bb0145] Naeem F., Phiri P., Nasar A., Gerada A., Munshi T., Ayub M., Rathod S. (2016). An evidence-based framework for cultural adaptation of Cognitive Behaviour Therapy: process, methodology and foci of adaptation. World Cult.Psychiatry Res.Rev..

[bb0150] National Collaborating Centre for Mental Health (2021). The improving access to psychological therapies manual. https://www.england.nhs.uk/publication/the-improving-access-to-psychological-therapies-manual/.

[bb0155] NICE (2013).

[bb0160] Oxford Centre for Anxiety Disorders and Trauma (2019). OXCADAT Resources: Resources for cognitive therapy for PTSD, social anxiety disorder and panic disorder. https://oxcadatresources.com/.

[bb0165] Pinheiro J., Bates D., DebRoy S., Sarkar D., R Core Team (2018). nlme: Linear and Nonlinear Mixed Effects Models. [R package version 3.1-131.1.]. https://CRAN.R-project.org/package=nlme.

[bb0170] R Core Team (2017). https://www.R-project.org/.

[bb0175] Rathod S., Phiri P., Naeem F. (2019). An evidence-based framework to culturally adapt cognitive behaviour therapy. Cogn.Behav.Ther..

[bb0180] Revelle W. (2018).

[bb0185] Rozental A., Andersson G., Boettcher J., Ebert D.D., Cuijpers P., Knaevelsrud C., Carlbring P. (2014). Consensus statement on defining and measuring negative effects of internet interventions. Internet Interv..

[bb0190] Selker R., Love J., Dropmann D. (2018). jmv: The 'jamovi' analyses. [R package version 0.8.6.2]. https://CRAN.R-project.org/package=jmv.

[bb0195] Spitzer R.L., Kroenke K., Williams J.B., Löwe B. (2006). A brief measure for assessing generalized anxiety disorder: the GAD-7. Arch. Intern. Med..

[bb0200] Stott R., Wild J., Grey N., Liness S., Warnock-Parkes E., Commins S., Clark D.M. (2013). Internet-delivered cognitive therapy for social anxiety disorder: a development pilot series. Behav. Cogn. Psychother..

[bb0205] Thew G.R. (2020). IAPT and the internet: the current and future role of therapist-guided internet interventions within routine care settings. Cogn.Behav.Ther..

[bb0210] Thew G.R., Ehlers A., Grey N., Wild J., Warnock-Parkes E., Dawson R.L., Clark D.M. (2020). Change processes in cognitive therapy for social anxiety disorder delivered in routine clinical practice. <sb:contribution><sb:title>Clin. Psychol.</sb:title> </sb:contribution><sb:host><sb:issue><sb:series><sb:title>Eur.</sb:title></sb:series></sb:issue></sb:host>.

[bb0215] Thew G.R., Powell C.L.Y.M., Kwok A.P.L., Lissillour Chan M.H., Wild J., Warnock-Parkes E., Clark D.M. (2019). Internet-based cognitive therapy for social anxiety disorder in Hong Kong: therapist training and dissemination case series. JMIR Form.Res..

[bb0220] Tracey T.J., Kokotovic A.M. (1989). Factor structure of the working alliance inventory. Psychol.Assess..

[bb0225] Tulbure B.T., Szentagotai A., David O., Ștefan S., Månsson K.N., David D., Andersson G. (2015). Internet-delivered cognitive-behavioral therapy for social anxiety disorder in Romania: a randomized controlled trial. PloS One.

[bb0230] Warnock-Parkes E., Wild J., Thew G.R., Kerr A., Grey N., Stott R., Clark D.M. (2020). Treating social anxiety disorder remotely with cognitive therapy. Cogn.Behav.Ther..

[bb0235] Watson D., Friend R. (1969). Measurement of social-evaluative anxiety. J. Consult. Clin. Psychol..

[bb0240] Wickham H. (2017). tidyverse: Easily Install and Load the 'Tidyverse'. [R package version 1.2.1]. https://CRAN.R-project.org/package=tidyverse.

[bb0245] Zimmerman M., Mattia J.I. (2001). A self-report scale to help make psychiatric diagnoses: the Psychiatric Diagnostic Screening Questionnaire. Arch. Gen. Psychiatry.

