# Supplementary material for: Internet-delivered cognitive therapy for social anxiety disorder in Hong Kong: A randomized controlled trial
Source: Internet Interv. 2022 Apr 18;28:100539. doi: 10.1016/j.invent.2022.100539 (PMC9046639; doi:10.1016/j.invent.2022.100539)
Supplement: Supplementary file 1 — Supplementary material [file mmc1.docx]

# Internet-based Cognitive Therapy for Social Anxiety Disorder in Hong Kong: A randomised controlled trial

**Supplementary Material**

**Generalised Learning Questionnaire (GLQ)**

The GLQ is shown below. This is an original scale developed for this study. An initial item pool was generated based on the findings from Thew et al. (in preparation), which was reviewed and refined on an iterative basis in conjunction with experts in the delivery and supervision of CT-SAD and iCT-SAD. Each item is rated on a 7-point Likert scale (“Totally agree”, “Agree very much”, “Agree slightly”, “Neutral”, “Disagree slightly”, “Disagree very much”, or “Totally disagree”). Responses are scored from 0 (totally disagree) to 6 (totally agree), with higher scores indicating greater generalised learning.

**GLQ**

Please indicate how much you agree with each statement:

1. **If one person I speak to finds me friendly and interesting, it is likely other people will too.**

| Totally  agree | Agree  very much | Agree  slightly | Neutral | Disagree slightly | Disagree very much | Totally disagree |
| --- | --- | --- | --- | --- | --- | --- |

1. **When people respond positively to me it is because I am a likeable person.**

| Totally  agree | Agree  very much | Agree  slightly | Neutral | Disagree slightly | Disagree very much | Totally disagree |
| --- | --- | --- | --- | --- | --- | --- |

1. **When people are looking at me, it is unlikely they are judging me negatively.**

| Totally  agree | Agree  very much | Agree  slightly | Neutral | Disagree slightly | Disagree very much | Totally disagree |
| --- | --- | --- | --- | --- | --- | --- |

1. **If people notice I am anxious, they are unlikely to think any less of me as a person.**

| Totally  agree | Agree  very much | Agree  slightly | Neutral | Disagree slightly | Disagree very much | Totally disagree |
| --- | --- | --- | --- | --- | --- | --- |

1. **When I am nervous, it is unlikely that I look as nervous as I feel.**

| Totally  agree | Agree  very much | Agree  slightly | Neutral | Disagree slightly | Disagree very much | Totally disagree |
| --- | --- | --- | --- | --- | --- | --- |

**Reference:**

Thew, G. R., Ehlers, A., & Clark, D. M. (in preparation). Sudden gains in face-to-face and internet-based Cognitive Therapy for Social Anxiety Disorder.
